# Dynamic community detection using class preserving time series generation with Fourier Markov diffusion

**DOI:** 10.1038/s41598-026-37699-1

**Published:** 2026-01-30

**Authors:** Yanfei Ma, Daozheng Qu, Yibo Wang

**Affiliations:** 1 Department of Computer Science, Fairleigh Dickinson University, Vancouver, V6B 2P6 Canada; 2 Department of Computer Science, University of Liverpool, Liverpool, L69 3DR UK; 3 Rady School of Management, University of California San Diego, San Diego, 92093 USA

**Keywords:** Dynamic community detection, Fake news cascades, Temporal graph networks, Reinforcement learning, Social media fake news, Computational biology and bioinformatics, Mathematics and computing

## Abstract

Generating class-consistent time series necessitates the maintenance of both overarching structure and detailed temporal dynamics–an endeavor that current GAN and diffusion models find challenging. We introduce FMD-GAN, a Fourier–Markov diffusion framework that integrates spectral clustering, state-conditioned frequency-domain noise modulation, and a dual-branch temporal–spectral discriminator to generate realistic and class-consistent sequences. In four UCR datasets (ECG200, GunPoint, FordA, ChlorineConc), FMD-GAN attains state-of-the-art or competitive outcomes, with up to a 50% reduction in FID and consistent enhancements in DTW, class consistency accuracy (CCA), and spectral distance (SD) compared to six representative baselines. Ablation studies validate the roles of spectrum masking, Markov-guided diffusion, and adversarial learning, whilst sensitivity analysis illustrates resilience to hyperparameters. Qualitative visualizations demonstrate significant semantic congruence between actual and produced samples. These findings indicate that the integration of spectral priors with probabilistic diffusion facilitates the production of time series that preserve structure and are cognizant of class distinctions, pertinent to biomedical monitoring, sensor analytics, and Tiny AI systems.

## Introduction

Applications in data augmentation^1,2^, simulation^3^, anomaly detection, biomedical signal synthesis^4^ and resource-constrained AI scenarios (e.g., Tiny AI, IoT devices) ^5^ are all supported by time series generation, a fundamental task in machine learning. Synthesizing realistic sequences that capture semantic structure and temporal connections is the aim. However, producing time series that are both semantically and structurally consistent remains challenging, especially in domains with complex latent dynamics such as physiological monitoring and human activity detection.

Advances in time-series generation employing foundation and transformer-based designs have been examined in recent surveys^6,7^. For better long-range forecasting, transformer variations like Autoformer^8^ use decomposition and auto-correlation techniques. Even while these techniques perform remarkably well in sequence modeling and forecasting, they frequently put realism or prediction accuracy ahead of interpretability and consistency across classes. The majority of current methods, in example, handle time series as undifferentiated temporal vectors without explicitly modeling frequency patterns or regime transitions, which restricts their use in contexts where structural control and semantic accuracy are crucial.

We provide **FMD-GAN**, a Frequency–Markov Diffusion Generative Adversarial Network that is intended to synthesize time series with both structural integrity and class consistency in order to overcome these constraints. FMD-GAN is motivated by the need to combine structural priors–such as frequency decomposition and latent regime modeling–with semantic awareness to enable interpretable and controllable generation.

Our system comprises three essential elements: frequency-aware denoising in a conditional diffusion process, Markov modeling of latent state transitions, and frequency-domain segmentation by spectral clustering. Stable and high-quality synthesis is made possible by score-based generative models^9^, which provide a rigorous method of training diffusion using stochastic differential equations (SDEs). By utilizing the interpretability of symbolic state modeling^10^, the expressiveness of conditional diffusion^11,12^, and the compactness of Fourier-based representations^13^, FMD-GAN breaks down sequences into spectral regimes and applies class-conditioned diffusion guided by latent states. The methodology works well for conditional generation and structure-sensitive data augmentation since it also guarantees that created samples stay semantically aligned with their targets through a class-consistency loss.

We test FMD-GAN on four exemplary datasets from the UCR Time Series Archive, which span different domains and sequence lengths: *ECG200*, *GunPoint*, *FordA*, and *UWaveGestureLibrary_X*. FMD-GAN achieves competitive or superior performance across multiple metrics, including Fréchet Inception Distance (FID), Dynamic Time Warping (DTW), Class Consistency Accuracy (CCA), and Spectral Distance (SD), when compared to six state-of-the-art baselines, including GAN-based, conditional, and diffusion models. Our framework’s interpretability, robustness, and semantic coherence are further illustrated by extensive qualitative evaluations, which include t-SNE projections, residual maps, latent state overlays, and training dynamics.

Our main contributions are as follows:

- We present **FMD-GAN**, a cohesive generative framework that amalgamates spectral clustering, Markov-guided latent modeling, and frequency-aware diffusion. Unlike current GAN-based models (e.g., TimeGAN, RCGAN-UCR) and diffusion-based models (e.g., DiffWave, Diffusion-TS), our system introduces a state-conditioned spectral noise mechanism, in which each Markov state defines a distinct Fourier-domain mask that parameterizes a non-isotropic diffusion covariance. This structured noise formulation transcends mere integration of existing modules, facilitating class-consistent and structure-preserving production influenced by regime-dependent spectral structure.
- We conduct comprehensive experiments on four UCR datasets and demonstrate that FMD-GAN attains performance that is either equivalent to or exceeds that of six representative baselines across many measures, including FID, DTW, class consistency accuracy (CCA), and spectral distance (SD). These results illustrate distinct advantages over both time-domain GANs and contemporary diffusion-based generators that do not incorporate state-aware spectral modeling.
- We perform thorough interpretability evaluations utilizing t-SNE projections, residual maps, and latent state overlays. The findings demonstrate that FMD-GAN acquires more coherent spectral regimes and class-discriminative latent structures compared to previous generative models, enhancing transparency and explainability.

Our framework transcends the mere integration of existing Fourier and Markov modules; the introduced state-conditioned spectral covariance–where each Markov state generates a unique non-isotropic diffusion noise profile–represents a structurally innovative mechanism not found in contemporary time-series GAN or diffusion models. The Fourier-Markov coupling enables FMD-GAN to more effectively maintain class semantics and regime-dependent dynamics, while also creating new opportunities for lightweight and interpretable signal generation in resource-limited environments.

## Related work

### Classical time series forecasting methods

Prior to the emergence of deep generative models, traditional statistical methods were predominantly employed for time series modeling and forecasting. Autoregressive Integrated Moving Average (ARIMA)^14^ and its seasonal versions have historically served as the principal methodologies for analyzing linear trends and seasonal patterns. State-space models, including the Kalman filter^15^ and Hidden Markov Models (HMMs)^10^, offer probabilistic frameworks that encapsulate temporal dependencies and regime transitions. Exponential smoothing approaches, such as Holt-Winters techniques^16^, facilitated effective forecasting across several industrial applications. Notwithstanding their efficacy in interpretability and efficiency, these methodologies frequently presuppose linearity, stationarity, or constrained dependence structures, hence limiting their capacity to simulate the nonlinear dynamics and intricate variability seen in contemporary high-dimensional time series. The aforementioned restrictions have spurred the investigation of deep learning-based generative frameworks, like GANs and diffusion models, to capture more intricate temporal patterns and generate realistic synthetic sequences.

### GAN-based models for time series generation

Generative adversarial networks (GANs) are extensively utilized for time series generation because of their capacity to model intricate distributions. C-RNN-GAN^17^ was the initial framework to adapt GANs for sequential data through the utilization of recurrent architectures. TimeGAN^1^ further implemented supervised embedding alignment to guarantee temporal and semantic accuracy. RCGAN-UCR^3^ integrated class-conditional methods to improve discriminability. Despite their achievements in short-range realism, GAN-based models frequently experience instability, a deficiency in interpretability, and a constrained capacity to capture long-term structure^18^.

### Diffusion models for temporal generation

Diffusion-based generative models have recently garnered attention for their resilience and sampling consistency, especially in time-series domains^19^. Score-based Stochastic Differential Equation frameworks and denoising diffusion probabilistic models provide well-founded training objectives and controllable generation. In the time-series domain, CSDI^20^ utilized conditional score matching for imputation, whilst Autoregressive DDPMs^11^ facilitate sequence-level conditioning. DiffWave^12^, Diffusion-TS^21^, and SigDiffusions^22^ aim to achieve high-fidelity signal generation for speech and physiological data. However, most of these models lack class-conditioning and overlook discontinuous latent transitions, which limits their semantic control and interpretability.

### Class-conditional and structured sequence models

Conditioning mechanisms for regulating the semantics of generated sequences have been the subject of numerous studies. Sequence-level conditioning in DDPMs enhances label fidelity^11^, while class-aware GANs^23^ and conditional VAEs^24^ allow label-guided generation. In parallel, interpretable temporal transitions are provided by symbolic models such as HMMs^10^. However, the majority of current frameworks do not incorporate symbolic state modeling into end-to-end diffusion processes.

### Hybrid models with semantic and structural constraints

A condensed and comprehensible viewpoint for capturing global temporal trends is provided by frequency-domain modeling. While neural Fourier operators^25^ have been used to learn periodic and structured representations in time series data, informer^13^ introduced spectral attention for long-range forecasting. These methods emphasize how crucial it is to use signal structure to enhance generalization.

To improve interpretability, robustness, and semantic control, recent surveys^6,7^ highlight the importance of integrating deep learning with symbolic priors, such as Markov segmentation and state transitions. In fields like physiological signal generation^26^, human motion modeling^27^, and dynamic system simulation^28^ that demand both high-fidelity synthesis and structural awareness, such hybrid approaches are especially pertinent.

However, in diffusion-based generative models, this hybrid approach is still not well studied. Our work advances this field by presenting **FMD-GAN**, a class-aware diffusion pipeline for semantically controllable and structurally faithful time series production that combines frequency-domain segmentation with Markovian latent transitions.

Hybrid designs that combine symbolic regime modeling with deep generative processes encounter significant hurdles, notwithstanding their theoretical attractiveness. Regime discovery typically depends on data-driven segmentation or clustering, whereas probabilistic state transitions impose modeling assumptions that may sacrifice flexibility for stability. These limitations largely elucidate why current diffusion-based generators typically eschew explicit state modeling and instead depend on universally applied noise schedules. Our research investigates a viable and empirically substantiated implementation of a hybrid architecture in the context of class-preserving time series production.

Positioning relative to recent frequency-based models. Recent frequency-domain architectures such as Informer^13^, Neural Fourier Operators^25^, TimesNet^29^, FEDformer^30^, and more recent models such as FreDF^31^ and TimeMixer^32^ further demonstrate the effectiveness of Fourier structure for long-range forecasting and representation learning. Nonetheless, these methodologies do not incorporate reverse diffusion, latent sampling, or stochastic reconstruction, and so cannot operate as generative models for unconditional or class-conditional synthesis. Their application of Fourier priors markedly contrasts with generative diffusion: it functions deterministically, lacks a denoising trajectory, and does not account for uncertainty or sample diversity. In contrast, diffusion-based generators such as DiffWave^12^, CSDI^20^, and Diffusion-TS^21^ support iterative reverse sampling and thus constitute appropriate baselines for our generative setting. However, current diffusion models generally depend on isotropic or globally scaled noise and fail to include regime-dependent latent dynamics. FMD-GAN distinguishes itself from both categories by closely integrating Fourier-domain structure with Markovian latent transitions: each latent state generates a unique spectral mask that defines a non-isotropic diffusion covariance, facilitating class-consistent, regime-aware synthesis–a feature lacking in previous GAN or diffusion models. We emphasize that this distinction is primarily driven by modeling objectives rather than superiority claims: forecasting-oriented frequency models and generative diffusion models address fundamentally different problem settings.

In contrast to recent conditional score-based diffusion models that employ learnable or globally parameterized noise schedules^22^, FMD-GAN adopts a state-conditioned noise formulation. Each latent Markov state specifically delineates a unique spectral covariance in the Fourier domain, leading to non-isotropic and regime-dependent diffusion dynamics. Conditional diffusion approaches adjust noise levels using auxiliary conditioning factors, however they often maintain a consistent noise structure across different time regimes. Our methodology directly integrates latent state transitions with frequency-domain noise modulation, facilitating regime-aware control absent in current score-based or conditional diffusion models.

In terms of frequency decomposition, Fourier-domain spectral clustering is selected to yield a comprehensive and interpretable division of frequency regimes that corresponds seamlessly with Markovian state modeling. Although wavelet-based or adaptive frequency decompositions focus on localized time-frequency fluctuations, our aim is to extract regime-level spectral signatures that maintain stability across temporal segments, rendering Fourier clustering more appropriate for state-aware diffusion control^31^.

While the above discussion positions FMD-GAN within the broader design space of state-aware and frequency-modulated diffusion models, Table 1 focuses on representative diffusion-based generators that are commonly adopted as experimental baselines in time-series synthesis.

**Table 1:** High-level architectural comparison between FMD-GAN and representative diffusion-based time-series generators.

Method	Frequency-aware	State-aware	Noise modulation
DiffWave^12^	No	No	Global / isotropic
Diffusion-TS^21^	No	No	Learnable global schedule
FMD-GAN	Yes (Fourier)	Yes (Markov)	State-conditioned spectral

### AI in information processing and tiny AI

Concurrent with advancements in generative modeling, there has been a growing focus on the role of artificial intelligence in effective information processing. Recent research underscores the necessity for compression, pruning, and quantization of deep neural networks to facilitate deployment on edge devices and microcontrollers^33,34^. The nascent realm of TinyML and on-device inference has highlighted the necessity of creating models that reconcile accuracy with efficiency, especially in areas like signal processing, IoT, and biomedical monitoring. These advancements indicate that progress in time-series creation must prioritize not only fidelity and interpretability but also deployability under resource limitations. Our suggested FMD-GAN, although primarily focused on class-consistent and structurally accurate generation, can be seamlessly adapted for efficient and lightweight applications.

## The proposed model

### Theoretical motivation

Time-series signals often exhibit two complementary forms of structure: (i) frequency-domain regularities such as periodicity and harmonic decay^13,29^, and (ii) regime-dependent temporal dynamics that evolve through discrete state transitions^35,36^. Standard diffusion models—whether applied in the temporal domain or directly to Fourier coefficients—inject isotropic Gaussian noise^37^ and therefore do not explicitly account for these state-dependent variations.

Our proposal integrates Fourier representations with a Markov-conditioned diffusion mechanism to close this gap. The Fourier-domain offers a concise structural prior, allowing us to configure the noise covariance through state-specific spectral masks that represent distinctive frequency patterns. Concurrently, latent states derived from spectral clustering progress via a first-order Markov chain, encapsulating regime-switching dynamics frequently found in time series^38^.

This interaction produces a non-stationary, state-aware diffusion trajectory that distinguishes itself from traditional diffusion models by tailoring noise injection to both spectral statistics and temporal state transitions. Consequently, the reverse process reconstructs signals that more effectively maintain global spectral form, local temporal dynamics, and class-dependent semantics.

To clarify why such a coupling is theoretically useful, we outline the complementary limitations of Fourier-only and Markov-only modeling in modern time-series analysis.

Recent Fourier-based architectures such as TimesNet^29^, FEDformer^30^, TimeMixer^32^, and FreDF^31^ demonstrate that spectral representations effectively capture global periodicity and long-range structure. However, these models typically depend on spatially fixed spectral assumptions, therefore possessing a constrained capacity to delineate the changing regime-dependent variability commonly observed in real-world signals. Although Fourier coefficients include global spectral information, they generally fail to represent the variations in spectral patterns across diverse temporal segments.

Conversely, regime-switching latent variable models–such as Structured State Space Models (SSMs)^36^ and Recurrent Switching Linear Dynamical Systems (rSLDS)^39^–provide a principled way to model discrete transitions between dynamical regimes. These methodologies function inside the time domain, and while they include temporal segmentation, they fail to explicitly delineate how uncertainty or variability should vary across frequency bands. Their covariance patterns are typically established across temporal dimensions, complicating the expression of frequency-selective stochasticity or spectrum fluctuations commonly associated with physiological or oscillatory signals.

The proposed Fourier–Markov coupling provides a method to connect these two viewpoints. Assigning a unique spectral mask to each latent state modulates the diffusion covariance in a frequency-dependent and state-aware fashion. This approach utilizes the Fourier-domain as a fundamental basis for articulating regime-specific variability, whereas the Markov chain regulates transitions between different regimes. Introducing noise in the Fourier-domain with state-dependent covariance produces a diffusion trajectory that is non-stationary and responsive to alterations in spectral structure. This combination does not seek to supplant Fourier-only or Markov-only models; instead, it offers an alternative generative framework that integrates aspects of both global spectral priors and regime-aware temporal dynamics.

The three fundamental elements of FMD-GAN–spectral clustering, Markov state transitions, and state-conditioned diffusion–are intended to function in a closely integrated manner rather than as separate heuristics. Spectral clustering divides the time series into latent regimes defined by unique frequency statistics, offering an intrinsic representation of regime-specific structure in the Fourier-domain. Modeling these regimes as latent Markov states encapsulates their temporal persistence and transition dynamics, frequently observed in real-world data. Conditioning the diffusion noise on these Markov states synchronizes uncertainty injection with regime-dependent spectral variability, allowing the diffusion process to modify its stochastic behavior in accordance with the underlying signal dynamics. This organized interaction boosts expressiveness by facilitating diverse yet coherent generation across several regimes, and improves stability by averting uncontrolled isotropic noise input that ignores regime structure.

The Fourier–Markov coupling serves as an auxiliary mechanism that allows FMD-GAN to condition its diffusion process on spectral structures based on different regimes. This approach differs from conventional DDPMs that depend on isotropic noise^37^, as well as from current frequency-only or state-only models that fail to incorporate spectral variability with temporal transitions.

### Architecture overview

**Fig. 1:**
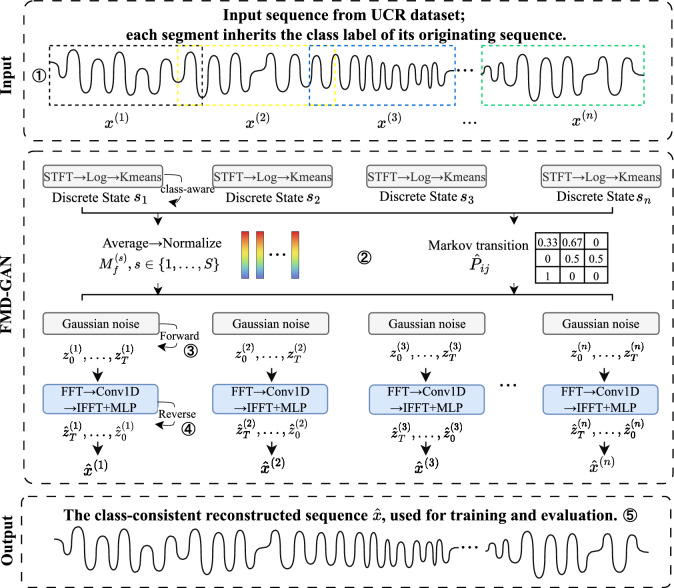
Overview of the proposed FMD-GAN architecture. The framework consists of five stages: (1) sliding-window segmentation of the input time series, (2) class-aware spectral clustering for latent state assignment, (3) Markov-guided forward diffusion with state-conditioned spectral noise, (4) reverse denoising and reconstruction conditioned on the latent state, and (5) overlap-aware aggregation to produce the final class-consistent sequence.

The suggested **Fourier–Markov Diffusion GAN (FMD-GAN)** architecture is described in this section. It uses frequency-domain noise modulation and class-aware latent states to produce realistic and semantically coherent time series. As shown in Fig. 1, the model comprises five main stages: sliding-window segmentation, class-guided state assignment, forward diffusion with state-conditioned noise, reverse generation, and dual-branch adversarial training.

We present a unique approach that combines latent state assignment and spectral clustering to guarantee class-consistent generation, allowing class-discriminative latent states to direct each time-series segment. In addition to controlling the forward diffusion process through frequency-domain masks, these states also condition adversarial learning and reverse generation, guaranteeing that the synthesized sequences match their original class labels semantically.

A complete pipeline is visualized in Fig. 1. Class-aware spectral clustering is used to divide each input time series into overlapping windows and assign a latent state. A Markov chain is used to simulate temporal transitions between latent states, and each state uses a learnt spectral mask to modify the forward diffusion noise. A reverse generator recovers the denoised output $\hat{\boldsymbol{x}}$ conditioned on the latent state, ensuring class-consistent reconstruction. A dual-branch discriminator is used to train the model under adversarial supervision, and its generation quality and class consistency are assessed.

### Sliding-window segmentation

Inspired by local receptive field strategies used in convolutional architectures^40^, we segment each input time series $\boldsymbol{x} \in \mathbb {R}^{L \times C}$ into overlapping sub-sequences using a sliding-window approach. Each sub-sequence $\boldsymbol{x}^{(n)} \in \mathbb {R}^{l \times C}$ is extracted with a fixed window length *l* and hop size *h*, where $n \in \{1, \dots , N\}$ indexes the window position. The total number of extracted windows is given by $N = \lfloor (L - l)/h \rfloor + 1$.

This method breaks down each long sequence into a set of fixed-size segments that serve as separate training examples for the subsequent generative modeling and spectral analysis stages.

### Class-aware state assignment via spectral features

Building on the windowed segments $\{\boldsymbol{x}^{(n)}\}_{n=1}^N$ obtained from the previous step, we now compute spectral features for each sub-sequence and assign class-aware latent states.

For each window $\boldsymbol{x}^{(n)}$, we compute the magnitude spectrum via the Short-Time Fourier Transform (STFT)^41^, where $\textrm{STFT}(\cdot )$ denotes the discrete short-time Fourier transform operator applied along the temporal dimension of each channel:

1 $$\begin{aligned} \boldsymbol{X}_f^{(n)} = \left| \textrm{STFT}\left( \boldsymbol{x}^{(n)} \right) \right| \in \mathbb {R}^{K \times C}, \end{aligned}$$

where $\boldsymbol{x}^{(n)}$ is the *n*-th windowed segment ($n=1,\dots ,N$, with *N* total windows from a sequence of length *L*), *K* is the number of frequency bins, and *C* is the number of channels. The magnitudes are logarithmically converted^18^ and aggregated across all windows to create a global spectral feature matrix.

**Fig. 2:**
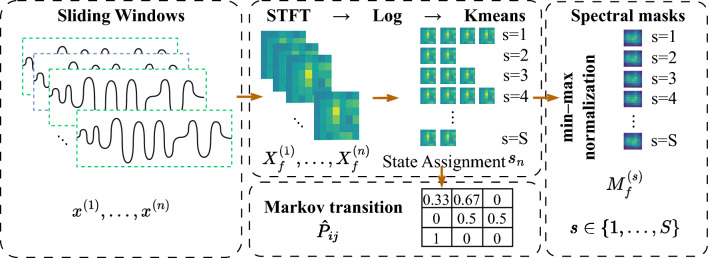
Overview of the class-aware state assignment pipeline. The input time series is first segmented into overlapping windows $\{\boldsymbol{x}^{(n)}\}$. Each window is transformed via STFT to obtain the magnitude spectrogram $\boldsymbol{X}_f^{(n)}$, followed by logarithmic scaling. The resulting spectral features are clustered using *k*-means to assign latent states $s_n$, which are aligned with class labels through majority voting. A Markov transition matrix $\hat{P}_{ij}$ is then estimated from the state sequence. Finally, a state-specific spectral mask $M_f^{(s)}$ is constructed by averaging and min–max normalizing the spectrograms of windows assigned to each state. Each spectral mask summarizes the averaged and normalized frequency-domain statistics of windows assigned to a latent state and is later used to parameterize state-conditioned diffusion noise.

An overview of this procedure is illustrated in Fig. 2, which summarizes the key stages from segmentation to state assignment, spectral mask construction, and transition modeling. The input time series is segmented into overlapping windows $\{\boldsymbol{x}^{(n)}\}$. Each window is transformed via STFT to obtain the magnitude spectrogram $\boldsymbol{X}_f^{(n)}$, followed by logarithmic scaling. Spectral features are clustered via *k*-means to assign latent states $s_n$, aligned with class labels through majority voting. A Markov transition matrix $\hat{P}_{ij}$ is estimated from the sequence of states. Additionally, each state’s spectral mask $M_f^{(s)}$ is computed by averaging the spectrograms of all windows assigned to that state and applying min–max normalization.

Let $s_n \in \{1,\dots ,S\}$ denote the discrete latent Markov state assigned to the *n*-th windowed segment, where *S* is the total number of latent states. To promote class consistency during generation, we cluster the log-spectrum of each window using $k$-means^42^, and align the resulting clusters with the ground-truth class labels:

2 $$\begin{aligned} s_n = \textrm{kmeans}\left( \log \boldsymbol{X}_f^{(n)} \right) \in \{1, \dots , S\}, \end{aligned}$$

where $\log \boldsymbol{X}_f^{(n)}$ signifies the log-scaled magnitude spectrum for numerical stability and feature improvement. When class labels are accessible, each cluster is aligned with the predominant class through majority voting on window-to-class associations, which indirectly promotes state assignments to represent class-discriminative features.

The temporal transitions between adjacent latent states yield the empirical Markov transition matrix^10^:

3 $$\begin{aligned} \hat{P}_{ij} = \Pr (s_{n+1} = j \mid s_n = i), \end{aligned}$$

where $\hat{P}_{ij}$ is the empirical probability of transitioning from state *i* to state *j*, with $i,j \in \{1,\dots ,S\}$, which captures the class-aware temporal structure within the dataset.

Finally, we group all windows by their assigned state $s \in \{1, \dots , S\}$, average their STFT magnitudes, and apply min–max normalization across frequency bins to construct a state-specific spectral mask:

4 $$\begin{aligned} M_f^{(s)} = \frac{1}{|\mathscr {W}_s|} \sum _{n \in \mathscr {W}_s} \boldsymbol{X}_f^{(n)}, \quad M_f^{(s)} \leftarrow \frac{M_f^{(s)} - \min M_f^{(s)}}{\max M_f^{(s)} - \min M_f^{(s)}}, \end{aligned}$$

where $\mathscr {W}_s = \{n: s_n = s\}$ is the set of windows assigned to state *s*, $|\mathscr {W}_s|$ its cardinality, and $\min (\cdot )$ and $\max (\cdot )$ denote the minimum and maximum values across frequency bins. This procedure yields a bank of spectral masks $\{M_f^{(s)}\}_{s=1}^S$, each reflecting the characteristic frequency distribution of its corresponding latent state. These masks are later used to modulate frequency-domain noise during forward diffusion, enabling class-sensitive perturbation. Normalization ensures that each mask defines a valid variance template bounded in [0, 1], suitable for stochastic noise control^43^.

### Fourier–Markov diffusion with state-conditioned noise

The forward diffusion process begins with an initial latent vector $\boldsymbol{z}_0 \sim \mathscr {N}(\boldsymbol{0}, \boldsymbol{I})$ for each segmented input. At every diffusion step $t = 0, 1, \dots , T{-}1$, the latent representation $\boldsymbol{z}_t$ is gradually perturbed with class-aware, state-conditioned Gaussian noise, modulated in the frequency domain.

In our concept, Markov conditioning is implemented at the level of the latent state designated to each spectral window, rather than on raw temporal segments or individual Fourier coefficients. These latent states are obtained by STFT-based spectral clustering, indicating that each state aligns with a coherent spectral range.

Specifically, at each step, a spectral noise vector $\boldsymbol{\varepsilon }_f$ is sampled from a zero-mean Gaussian distribution with a diagonal covariance matrix shaped by a state-specific spectral mask:

5 $$\begin{aligned} \boldsymbol{\varepsilon }_f \sim \mathscr {N}(\boldsymbol{0}, M_f^{(s_n)} \odot \boldsymbol{I}), \end{aligned}$$

where $M_f^{(s_n)} \in [0,1]^K$ is the Fourier-domain mask corresponding to the current Markov state $s_n$, which parameterizes the diagonal covariance of the injected frequency-domain noise, and $\odot$ denotes elementwise multiplication. By encoding class-discriminative spectral patterns, these masks guarantee that injected noise preserves the original class’s semantic structure. This technique promotes class-consistent generative routes over time by encouraging the diffusion trajectory to stay in line with class semantics.

The latent is then updated as:

6 $$\begin{aligned} \boldsymbol{z}_{t+1} = \sqrt{\alpha _t}\,\boldsymbol{z}_t + \sqrt{1 - \alpha _t}\,\boldsymbol{\varepsilon }_f, \end{aligned}$$

where $\{\alpha _t\}$ is a linear variance schedule controlling the noise scale at each step.

Across adjacent windows, latent states evolve according to the Markov prior $P(s_{n+1} \mid s_n)$. During diffusion for a given window *n*, the state $s_n$ remains fixed across all diffusion steps $t=0,\dots ,T-1$. The estimate of the state transition matrix over class-aware spectral clusters simulates temporal transitions in this stochastic process while preserving label-consistent fluctuations.

The transition rule adheres to a **first-order Markov assumption**, wherein each state is contingent solely upon its immediate predecessor. Despite the explicit model being first-order, higher-order temporal relationships are implicitly encapsulated by overlapping windows, non-stationary spectral masks, and the cumulative architecture of the reverse diffusion trajectory.

The model integrates both controlled variability and structural consistency by conditioning frequency-domain perturbations on latent states that evolve according to a Markov process and encapsulate class semantics. This ensures that the diffusion trajectory remains in line with class-specific dynamics that are seen in sequences from the real world.

The same window-level state assignment $s_n$ is used to condition both the forward and reverse processes for window *n*, ensuring consistent state-aware generation.

### Reverse generation and segment aggregation

The reverse generator $G_\theta$ reconstructs the class-consistent latent vector $\hat{\boldsymbol{z}}_0$ from a heavily perturbed latent $\boldsymbol{z}_T$ by progressively denoising it through $T$ steps. At each reverse step $t = T{-}1, \dots , 0$, the model learns to approximate the class-aware conditional distribution:

7 $$\begin{aligned} p_\theta (\boldsymbol{z}_t \mid \boldsymbol{z}_{t+1}, s_n, t) \end{aligned}$$

where $s_n$ is the Markov state sampled during the forward process. State transitions encapsulate spectral patterns that correspond with class labels, hence each reversal step is directed by a semantically significant structure.

Here, $\textrm{FFT}(\cdot )$ and $\textrm{IFFT}(\cdot )$ denote the discrete Fourier transform and its inverse, respectively, applied along the temporal dimension of each channel. The reverse generator follows a hybrid spectral–temporal procedure:

8 $$\begin{aligned} \boldsymbol{Z}_{t+1} = \textrm{FFT}(\boldsymbol{z}_{t+1}), \end{aligned}$$

where each channel’s temporal dimension is subjected to a fixed-size 1D FFT. To guarantee constant spectral resolution across all steps, we employ zero-padding for segments that are smaller than the FFT size.

9 $$\begin{aligned} \boldsymbol{Z}_{t+1}^{\text {filt}} = \textrm{Conv1D}(\boldsymbol{Z}_{t+1}; \phi (s_n)), \end{aligned}$$

10 $$\begin{aligned} \hat{\boldsymbol{z}}_t = \textrm{IFFT}(\boldsymbol{Z}_{t+1}^{\text {filt}}) + \textrm{MLP}(t), \end{aligned}$$

11 $$\begin{aligned} \hat{\boldsymbol{z}}_t \leftarrow \gamma (s_n) \cdot \hat{\boldsymbol{z}}_t + \beta (s_n), \end{aligned}$$

where $\phi (s_n)$ denotes a state-conditioned convolutional filter applied in the frequency domain, and $\gamma (\cdot ), \beta (\cdot )$ are FiLM^44^ parameters generated from state embeddings. These layers function as class-sensitive modulators, enabling the generator to modify the denoising trajectory according to latent class attributes.

At the end of the process, the cleaned latent vector $\hat{\boldsymbol{z}}_0$ is decoded into a window-level time series segment:

12 $$\begin{aligned} \hat{\boldsymbol{x}}^{(n)} = \textrm{Dec}_\theta (\hat{\boldsymbol{z}}_0). \end{aligned}$$

Here, $\hat{\boldsymbol{x}}^{(n)}$ represents the reconstructed segment of the $n$-th window. These segments are later aggregated to form the full-length synthetic sequence $\hat{\boldsymbol{x}}$, which preserves both the structural variation and the semantic class identity of the original data.

After denoising each latent segment via the reverse process, the generator produces a set of window-level reconstructions $\{\hat{\boldsymbol{x}}^{(n)}\}_{n=1}^{N}$. To obtain the final sequence $\hat{\boldsymbol{x}} \in \mathbb {R}^{L \times C}$, these overlapping segments are aggregated into a coherent time series through an overlap-aware stitching strategy^45^, similar to the classic overlap-add technique in STFT reconstruction.

Given a fixed hop size $h < w$, where $w$ is the segment/window length, overlapping regions are averaged to ensure temporal smoothness and reduce boundary artifacts. For each time step $l \in [1, L]$, the reconstructed value is computed by:

13 $$\begin{aligned} \hat{\boldsymbol{x}}[l] = \frac{1}{|\mathscr {N}_l|} \sum _{n \in \mathscr {N}_l} \hat{\boldsymbol{x}}^{(n)}[l - o_n], \end{aligned}$$

where $\mathscr {N}_l$ is the set of windows covering position $l$, and $o_n = (n - 1) \cdot h$ is the offset of the $n$-th window.

This aggregation technique maintains class-discriminative local patterns within each segment while enhancing temporal continuity in the reconstructed sequence. The produced signals are appropriate for auxiliary applications, like data augmentation, qualitative analysis, or controlled review, without suggesting enhancements in downstream task performance.

The reconstructed sequence is then used in all evaluation scenarios and fed into a dual-branch discriminator during adversarial training.

### Adversarial training with class-aware dual-branch discriminator

We use a class-aware dual-branch discriminator $D_\phi$ and the WGAN-GP framework^46^ to synthesize realistic and class-consistent time series. Working with the entire reconstructed sequence $\hat{\boldsymbol{x}}$, the discriminator gives the generator adversarial feedback that directs it to replicate both class-specific temporal dynamics and global structure.

**Fig. 3:**
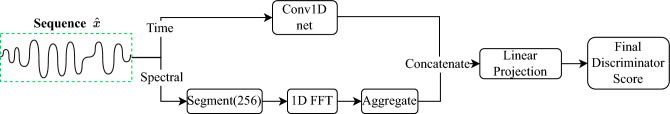
FMD-GAN’s class-aware dual-branch discriminator architecture. The temporal branch utilizes a Conv1D network to evaluate local waveform authenticity and short-term dynamics, whilst the spectral branch analyzes non-overlapping windows of 256 samples with a 1D FFT and consolidates magnitude spectra to encapsulate global frequency characteristics. Features from both branches are concatenated and subjected to a linear projection to get the final discriminator score, simultaneously enforcing temporal continuity and spectral coherence.

Two parallel branches make up the discriminator, as seen in Fig. 3. The **time branch** evaluates local signal coherence and temporal continuity using a 1D convolutional network. The **spectral branch** applies a fixed-size 1D FFT to each channel of $\hat{\boldsymbol{x}}$ in order to assess holistic frequency-domain features. In order to improve numerical stability and highlight informative frequency patterns like rhythm or repetition, log-magnitude scaling ($\log (1 + |\cdot |)$ is employed. The spectral branch divides each full-length input into non-overlapping windows of length 256 in order to guarantee constant frequency resolution over sequences of different lengths. A global spectral representation is created by averaging the magnitude spectra obtained from a 1D FFT of each window. The discriminator can capture long-range spectral structure while keeping a stable frequency bin size ($K=129$) across datasets thanks to this aggregation method. Because there is no windowing or framing, global spectral properties are preserved.

The two branches serve unique yet complementary aims. The time-domain branch functions as a temporal evaluator, assessing short-term waveform authenticity, transitions, and intricate temporal changes specific to each class. Conversely, the spectral branch operates as a *structural critic*, ensuring the maintenance of global harmonic patterns, prevailing frequencies, and the overall spectral envelope. Consequently, the dual-branch architecture explicitly incorporates both time-domain and frequency-domain discriminators, allowing the model to concurrently evaluate temporal fidelity (semantics) and spectrum structure (global patterns). This method guarantees that generated sequences align with actual samples in their immediate temporal dynamics while also adhering to class-consistent spectral characteristics.

A scalar discriminator score is obtained by concatenating the outputs of both branches and passing them through a linear projection head. Both temporal fidelity and spectral coherence are reflected in this score, which allows $D_\phi$ to function as an auxiliary classifier that promotes class-consistent generation as well as a realism evaluator.

Through this dual-branch formulation, the discriminator provides richer and more disentangled feedback to the generator: (1) the temporal critic constrains semantic dynamics, and (2) the structural critic constrains frequency-domain consistency. This addresses the reviewer’s question by clarifying the distinct goals and complementary roles of the two branches.

To further preserve class-specific dynamics, we incorporate a **transition-level regularization** based on Markov state assignments. From training data, we estimate an empirical state transition matrix $\hat{P}$, capturing typical evolution patterns. During generation, a latent state sequence $\{s_1, \dots , s_N\}$ is obtained across adjacent windows, inducing a predicted transition matrix $P_\theta$. A KL divergence penalty is used to encourage consistency between $P_\theta$ and $\hat{P}$, promoting realistic intra-class transitions.

The final training objective integrates four components: an adversarial loss $L_{\text {adv}}$, a spectral reconstruction loss, a transition regularization term, and a latent reconstruction penalty. The overall loss is defined as:

14 $$\begin{aligned} L \;=\;&L_{\text {adv}} + \lambda _{\text {spec}} \bigl \Vert |\textrm{FFT}(\hat{\boldsymbol{x}})| -|\textrm{FFT}(\boldsymbol{x})| \bigr \Vert _2^{2} \nonumber \\&+ \lambda _{\text {KL}}\, \textrm{KL}\!\bigl (P_\theta \,\Vert \,\hat{P}\bigr ) + \lambda _{\text {rec}}\, \bigl \Vert \boldsymbol{z}_0 - \hat{\boldsymbol{z}}_0\bigr \Vert _2^{2}. \end{aligned}$$

The spectrum loss enforces frequency alignment, the KL term maintains temporal dynamics, and the reconstruction penalty guarantees successful reversal of the diffusion process. Collectively, these aims empower FMD-GAN to generate coherent, structurally accurate, and class-sensitive time series.

### Pseudocode of FMD-GAN training

We summarize the complete training workflow of FMD-GAN in Algorithm 1, which integrates spectral clustering, forward diffusion, reverse generation, and adversarial optimization.

**Algorithm 1:**
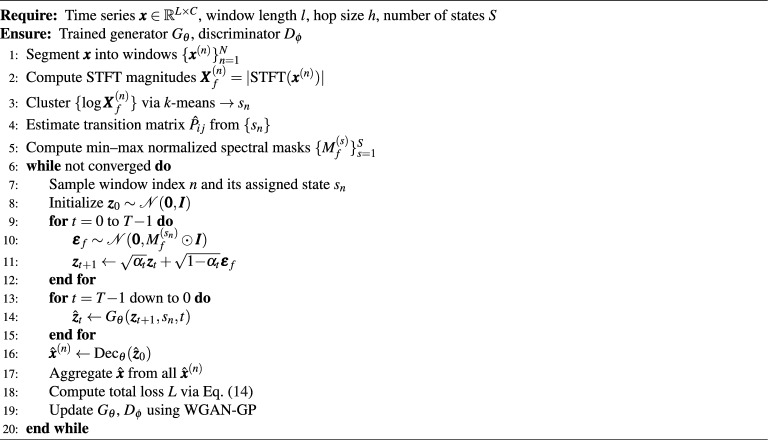
Training procedure of FMD-GAN.

### Computational complexity analysis

We now analyze the computational complexity of each stage in the proposed FMD-GAN framework. Let $L$ be the length of the input time series, $C$ the number of channels, $l$ the window length, $h$ the hop size, and $N = \lfloor (L - l)/h \rfloor + 1$ the number of segments per sequence. The segmentation process itself requires $\mathscr {O}(N \cdot l \cdot C)$ operations, as each window is extracted from the original sequence.

The class-aware state assignment involves computing the Short-Time Fourier Transform (STFT) for each segment, with a per-window cost of $\mathscr {O}(l \log l \cdot C)$, resulting in a total complexity of $\mathscr {O}(N \cdot l \log l \cdot C)$. The subsequent spectral clustering via $k$-means over log-magnitude spectra incurs $\mathscr {O}(I \cdot N \cdot K)$ complexity, where $I$ is the number of iterations and $K$ is the number of frequency bins.

During the forward diffusion stage, each latent segment is perturbed over $T$ steps. At each step, generating spectral noise and performing elementwise operations with the spectral mask requires $\mathscr {O}(K)$, yielding a total of $\mathscr {O}(T \cdot K)$ per segment. Given $N$ segments, the overall complexity of forward diffusion is $\mathscr {O}(N \cdot T \cdot K)$.

The reverse generation process applies a frequency-domain convolution using FFT-based filtering and FiLM modulation. Each FFT/IFFT pair has a complexity of $\mathscr {O}(K \log K)$, and the convolution and FiLM layers contribute an additional $\mathscr {O}(K)$. Across $T$ reverse steps and $N$ segments, the total reverse generation complexity is $\mathscr {O}(N \cdot T \cdot K \log K)$.

Segment aggregation involves averaging overlapping regions, with a total time proportional to the sequence length, $\mathscr {O}(L \cdot C)$. The dual-branch discriminator performs both temporal and spectral discrimination. The time-branch convolution operates in $\mathscr {O}(L \cdot C)$, while the spectral branch computes an FFT and MLP over the whole sequence, incurring $\mathscr {O}(L \log L \cdot C)$.

The total training difficulty per sequence per iteration is primarily determined by the STFT-based spectral clustering, the iterative forward and reverse diffusion processes, and the evaluation of the discriminator, culminating in an overall cost of:

15 $$\begin{aligned} \mathscr {O}(N \cdot l \log l \cdot C + I \cdot N \cdot K + N \cdot T \cdot K \log K + L \log L \cdot C) \end{aligned}$$

This complexity is manageable in reality, as the fundamental elements–such as segment-wise operations and frequency-domain transformations–are highly parallelizable. Furthermore, the implementation of FFT-based spectral modeling diminishes computational expenses relative to recurrent or attention-based methods, rendering FMD-GAN particularly appropriate for extensive time series.

Discrete diffusion and Markov transitions. FMD-GAN employs a discrete-time diffusion process with a fixed number of steps ($T=50$), following the DDPM family. Importantly, diffusion steps do not correspond to Markov transitions; each window retains a fixed latent state during the entire diffusion trajectory, while Markov transitions operate only across adjacent windows ($s_n \rightarrow s_{n+1}$). This design cleanly decouples per-window denoising from temporal state evolution.

Efficiency of the Fourier–Markov interaction. The interplay between Fourier-domain masks and Markov states is computationally efficient. All spectral masks and state assignments are precomputed and do not necessitate gradient updates; applying a mask is merely elementwise multiplication in the frequency domain.

Scalability to long sequences and large datasets. Each diffusion step requires a single FFT of cost $\mathscr {O}(K \log K)$, and overall runtime grows linearly with the number of windows and diffusion iterations. The parallelizable nature of spectral operations and the absence of runtime overhead from Markov transitions during training enable FMD-GAN to scale efficiently to extended sequences. We empirically found consistent training and almost linear computational growth as sequence length grew across UCR datasets.

### Training stability and parameter settings

To improve reproducibility, we present the justification for the essential parameter configurations of FMD-GAN. The sliding-window length was set at $l=64$ with a hop size of $h=16$, resulting in a 75% overlap between successive segments. This design ensures the retention of fine-grained temporal patterns while minimizing redundancy. This represents a practical balance between temporal resolution and computing expense: greater increments (e.g., $h=32$) overlook local dynamics and induce discontinuities, while smaller increments (e.g., $h=8$) result in duplicated samples and increased training costs. Empirically, $h=16$ resulted in a more refined reconstruction during overlap-add aggregation and diminished boundary artifacts, producing superior DTW and spectral distance (SD) scores compared to coarser alternatives.

The latent states were established at $S=8$; values below this threshold inadequately represented variety, while higher values resulted in unstable clustering results. The forward diffusion process was executed over $T=50$ iterations with a linear beta schedule. Initial experiments with $T \in [25, 100]$ demonstrated that $T=50$ achieves the best balance between generation fidelity and runtime efficiency.

FMD-GAN employs the WGAN-GP framework with gradient penalization for adversarial training, a well-established technique for stabilizing GAN optimization. Checkpoints were chosen based on the minimal validation Fréchet Inception Distance (FID), which also served as an early halting criterion to avert overfitting. These approaches collectively enhance training stability and ensure constant convergence.

Further implementation details, including optimizer configuration and architectural specifications, are reported in the Implementation Details section.

Training stability. Despite FMD-GAN’s simultaneous optimization of adversarial objectives, diffusion regularization, spectral alignment, and Markov-transition constraints, the overall training stability is maintained by several design elements. Initially, we implement the WGAN-GP framework, recognized for its substantial enhancement of generator-discriminator convergence and its mitigation of gradient explosion. Second, the diffusion component is constrained by well-balanced reconstruction, spectral, and KL losses ($\lambda _{\textrm{spec}}=1.0$, $\lambda _{\textrm{KL}}=0.1$, $\lambda _{\textrm{rec}}=1.0$), preventing conflict between competing objectives. Third, the Markov states and spectral masks are precomputed and remain constant throughout training, so avoiding extraneous sources of gradient noise. The frequency-domain branch consolidates FFT magnitudes across windows, yielding a smoothed and numerically stable discriminator signal. These methods collectively guarantee that optimization functions effectively and empirically converges without instability.

Practical guidance. The quantity of latent states *S* governs the granularity of regime modeling and may be augmented for datasets exhibiting more intricate or heterogeneous dynamics. The diffusion step count *T* significantly influences the fidelity-efficiency trade-off and can be modified for extended sequences, but at the expense of runtime. Other hyperparameters, including window length, FFT resolution, and loss weights, exhibited reduced sensitivity within acceptable ranges and are thus advised to remain at their default settings unless significant domain-specific limitations exist. We advise commencing with the default setup shown in this study and conducting minimal sensitivity analyses when transitioning to significantly different data environments. A pragmatic method involves adjusting *S* by observing clustering stability (e.g., variance of cluster assignments across seeds) and modifying *T* by incrementally increasing it until improvements in FID/DTW plateau under the same runtime constraints.

### Implementation considerations

Alongside stability analysis, we emphasize various design decisions of FMD-GAN that facilitate effective training and suggest possibility for lightweight implementation. The application of Fourier decomposition and Markovian state modeling minimizes redundancy by distinguishing frequency-specific regimes, hence alleviating the strain on the diffusion process relative to handling sequences as uniform temporal vectors. Secondly, the design utilizes fewer diffusion steps than traditional models, demonstrating that efficiency can be attained without sacrificing realism. The modular architecture of FMD-GAN facilitates integration with established model compression and acceleration methods, including pruning, quantization, and low-rank factorization^33,34,47^. These characteristics indicate that, in addition to methodological improvements in class-consistent time series creation, FMD-GAN can be modified for use in resource-limited settings such as Tiny AI and IoT devices.

## Experiment

We evaluate the effectiveness of FMD-GAN in the realm of class-preserving time series generation. Experiments are conducted using meticulously selected datasets from the UCR Time Series Archive^48^, covering diverse domains and sequence lengths.

### Datasets

We assess FMD-GAN using four representative datasets from the UCR Time Series Archive^48^, chosen to encompass a varied spectrum of sequence lengths, class quantities, and application fields. This diversity facilitates a thorough evaluation of the model’s generalization ability across different structural and semantic patterns.

**Table 2:** Summary of datasets used for evaluation.

Dataset	#Classes	Length	#Instances	Domain
ECG200	2	96	200	Biomedical
GunPoint	2	150	200	Human motion
FordA	2	500	1320	Industrial sensor
ChlorineConc	3	166	4307	Chemical process monitoring

Table 2 delineates the principal attributes of the chosen datasets. *ECG200* consists of brief univariate heartbeat impulses derived from electrocardiograms and is widely used as a benchmark for biomedical signal processing. *GunPoint* captures arm motion dynamics and serves as a canonical gesture recognition dataset in sensor-based human activity studies. *FordA* comprises extensive univariate sequences captured from engine sensors throughout various operating circumstances, making it a representative industrial anomaly detection dataset. *ChlorineConc* contains concentration measurements from a simulated chemical process, providing a challenging benchmark for multi-class monitoring and temporal structure modeling.

For each dataset, we utilize the official training and testing divisions supplied by UCR. During training, sequences are partitioned into overlapping sub-sequences utilizing a sliding window of length $l = 64$ and a hop size of $h = 16$. Every segment is normalized to a mean of zero and a variance of one. During inference, produced segments are recombined by overlap-aware averaging to recreate the complete time series for assessment.

*Note.* For the spectral-distance analysis in the Quantitative Results Section, we additionally report results on two compact UCR datasets (Coffee and Beef), which are widely used for evaluating frequency-structure preservation.

Cross-domain evaluation. We did not include cross-domain experiments (training on one UCR dataset and evaluating on a different one), because UCR datasets differ substantially in sampling rate, temporal scale, and class semantics, which makes direct cross-dataset generation ill-posed and difficult to interpret. In this work, we therefore focus on class-preserving generation within each dataset using its official train/test split. Nonetheless, the proposed Fourier-Markov design is domain-agnostic, and extending FMD-GAN to explicit cross-domain transfer remains an interesting direction for future research.

### Baselines

We compare FMD-GAN against six competitive generative baselines, each representing a distinct paradigm in time series generation:

- **TimeGAN**^1^ (Adversarial + Supervised): A hybrid model integrating RNN-based autoencoding, temporal supervision, and adversarial learning. It serves as a prevalent standard for sequential generation.
- **RCGAN-UCR**^3^ (Conditional GAN): A recurrent conditional GAN initially designed for the synthesis of medical signals. We modify it for UCR datasets by conditioning on one-hot class labels.
- **TTS-CGAN**^49^ (Prototype-guided GAN): A GAN model that produces time series by conditioning on class prototypes, hence improving semantic integrity and temporal coherence.
- **CSDI**^20^ (Score-based Diffusion): A conditional score-based diffusion model for imputing time series data. We adapt it for unconditional generation by class-aware reverse sampling.
- **DiffWave**^12^ (Denoising Diffusion): An audio synthesis diffusion model, modified for unconditional time series production with Gaussian noise schedules.
- **Diffusion-TS**^21^ (Denoising Diffusion): A comprehensible time series generator utilizing autoregressive denoising diffusion, providing high fidelity across many tasks.

To facilitate an equitable comparison, all baselines are trained on identical windowed and normalized sequences as FMD-GAN (see the Datasets Section), employing the same segment lengths, class conditioning procedures, and evaluation metrics. Hyperparameters are optimized through validation, and all models are assessed using identical splits.

These baselines provide a thorough assessment of FMD-GAN across adversarial, conditional, and diffusion-based frameworks, especially in structure-aware and class-preserving generation tasks.

It is observed that numerous contemporary frequency-decomposed time-series models are predominantly intended for forecasting or representation learning, rather than for stochastic sampling-based generation; thus, they are referenced in the Related Work section but are not directly comparable as generative baselines within our class-conditional synthesis framework.

Fairness and baseline adaptation. All baseline models were trained under the same experimental conditions as FMD-GAN to guarantee a fair comparison. All approaches employed an identical train/validation/test split, a consistent window length ($l=64$), a uniform hop size ($h=16$), and the same normalized inputs. For generative models requiring a latent code (e.g., TimeGAN, RCGAN, CSDI, DiffWave), the latent dimension was fixed to 64 to match the configuration of FMD-GAN. All models were trained using the Adam optimizer with identical learning rate ($1\times 10^{-4}$), batch size (64), and a maximum budget of 5000 iterations, with early stopping based on validation FID applied consistently to every baseline.

We modified the decoder of models initially intended for distinct domains (e.g., DiffWave for audio, CSDI for imputation) to produce complete sequences of length *L*, while maintaining the original architectural frameworks. Conditional models (TimeGAN, RCGAN, TTS-CGAN) were trained utilizing the identical one-hot labels employed by FMD-GAN. These adjustments ensure that performance variations are indicative of modeling proficiency rather than inconsistencies in training methodologies.

### Evaluation metrics

To thoroughly examine the quality of generated time series, we employ four representative metrics that together measure realism, structural fidelity, semantic consistency, and interpretability.

- **Fréchet Inception Distance (FID):** Evaluates the distributional similarity between authentic and produced samples inside a learned embedding space. We employ a pretrained LSTM encoder to derive fixed-length representations and calculate the Fréchet distance between the empirical Gaussian distributions of these embeddings. A reduced FID signifies enhanced distributional alignment and authenticity.
- **Dynamic Time Warping (DTW):** Determines structural alignment by calculating the best alignment cost between generated and actual sequences. Dynamic Time Warping accommodates local time variations and distortions, rendering it a resilient metric for temporal accuracy. Reduced DTW values signify enhanced structural preservation.
- **Class Consistency Accuracy (CCA):** Evaluates semantic fidelity by checking whether generated sequences remain consistent with their intended class labels. A 1D CNN classifier is trained only on real training data and then used to predict labels for generated samples. We employ CCA as a minimal proxy for downstream label fidelity; while it cannot replace task-specific deployment evaluation, it offers a controlled and model-agnostic measure of whether synthetic samples maintain class-discriminative patterns under a consistent decision rule. To facilitate an equitable comparison, identical classifier design, training protocols, and decision thresholds are employed across all methodologies, and the classifier is validated to attain satisfactory accuracy on the UCR held-out real test split prior to its application in CCA evaluation. An elevated CCA signifies superior class-conditional generation quality.
- **Spectral Distance (SD):** Assesses the preservation of frequency-domain structure by calculating the average Euclidean distance between the normalized power spectra of actual and produced sequences. We utilize the Fast Fourier Transform (FFT) to derive the magnitude spectrum for each sequence. Reduced SD values signify enhanced global structural alignment in the frequency domain, reinforcing the spectral modeling rationale underlying FMD-GAN. Rather than focusing on particular frequency bands in isolation, SD assesses the comprehensive spectral profile of a sequence. This architecture corresponds with our regime-aware modeling objective, wherein latent states reflect distinctive spectral patterns across many frequency ranges, rather than adhering to rigid band-wise limitations. Improvements in SD indicate the concurrent preservation of low-frequency structural elements and higher-frequency changes linked to each regime.

All quantitative measures are averaged across five independent trials with distinct random seeds to provide statistical robustness. For each evaluated method and metric, both the mean and standard deviation are reported consistently in the corresponding result tables. Classifiers employed for CCA and embeddings utilized for FID and t-SNE remain constant throughout all methodologies.

Statistical protocol. All experiments are conducted five times with distinct random seeds, and results are shown as mean ± standard deviation. Owing to the limited number of repeated trials and the computational expense associated with diffusion-based training, formal paired significance tests (e.g., Wilcoxon signed-rank or paired *t*-tests) were not performed. Reliability of the reported performance enhancements is evaluated through consistency across various seeds, convergence behavior in training dynamics, and complementing ablation investigations, adhering to established practices in generative modeling literature.

Rationale and interpretation of metrics. Each metric is incorporated to examine a distinct facet of class-preserving generation. FID functions as a metric for distribution-level realism inside a learnt feature space, encapsulating global sample quality beyond mere pointwise alignment. DTW enhances FID by directly assessing temporal alignment amidst local phase shifts, which is essential for time-series with potentially time-warped discriminative patterns. SD is incorporated to empirically evaluate our primary modeling assertion–that frequency-domain structure must be maintained–by contrasting normalized power spectra instead of time-domain trajectories. Ultimately, CCA serves as a task-oriented proxy for semantic fidelity: a classifier trained on authentic data predicts labels for created samples, determining if synthetic sequences are recognizable according to a predetermined downstream decision rule. We assert that CCA is not designed to supplant downstream task evaluation; rather, it offers a streamlined and model-agnostic measure of label consistency without the need for further task-specific training objectives.

### Implementation details

***Training setup.*** All models are executed in PyTorch 1.13 and trained on a solitary NVIDIA RTX 3090 GPU. Both datasets utilize a fixed window length of $l = 64$ and a hop size of $h = 16$ for time series segmentation. The quantity of latent states is established at $S = 8$, and the count of diffusion steps is predetermined at $T = 50$. A linear beta schedule is utilized for the forward diffusion process. The choice of $S=8$ is not a structural limitation of the framework; it was selected because it provided stable clustering and consistent performance across all UCR datasets. Larger values are supported by the model but tended to produce noisy or unstable clusters on these small datasets.

***Optimization.*** The Adam optimizer is employed with a learning rate of $1 \times 10^{-4}$, a batch size of 64, and no weight decay. Each model undergoes training for 5000 iterations, although empirical convergence generally occurs at approximately 3800 steps. The checkpoint exhibiting the minimal validation Fréchet Inception Distance (FID) is chosen for final assessment.

***Model architecture.*** The generator has three temporal convolutional layers (kernel size 5, dilations 1–2–4), succeeded by two linear projections to align with the input channel dimension $C$. Every diffusion step is influenced by a spectral mask $\boldsymbol{M}_f^{(s)} \in \mathbb {R}^{K}$, with $K = 129$ representing the quantity of positive frequency bins derived from a 256-point FFT. Zero-padding is utilized to guarantee that all segments conform to the FFT input dimensions.

Despite each input segment is 64 in length, we utilize a 256-point FFT to improve frequency resolution. The enhanced spectral granularity enables each mask $\boldsymbol{M}_f^{(s)}$ to identify more distinctive frequency patterns for each latent state, thus enhancing both structure-aware generation and spectral consistency. Empirical observations indicate that reduced FFT sizes result in diminished modularity and alignment in generated sequences.

The reverse generator replicates the forward pipeline and executes incremental denoising based on latent states. The discriminator functions at the full-sequence level and has a dual-branch architecture, simultaneously optimizing for adversarial realism and class-aware precision. It employs 1D FFT-based spectral feature extraction, succeeded by LeakyReLU activations and LayerNorm.

***Clustering and latent state assignment.*** We execute K-means clustering on the segment-level spectral embeddings to derive state labels for frequency masking. The cluster count is established at $S = 8$, with 100 iterations employed. To ensure numerical stability, we can employ PCA to reduce embeddings to 32 dimensions prior to clustering.

***Loss function and weighting.*** The full FMD-GAN model is trained using the unified objective defined in Eq. (14), which integrates adversarial learning with spectral alignment, latent-state regularization, and reconstruction consistency. Unless otherwise specified in ablation studies, all experiments use the weighting configuration $\lambda _{\text {rec}} = 1.0$, $\lambda _{\text {spec}} = 1.0$, and $\lambda _{\text {KL}} = 0.1$. The spectral loss quantifies the mean squared error between actual and generated magnitude spectra, whereas the KL loss regulates the distribution of assignments among latent states.

***Key hyperparameters summary.*** For clarity and reproducibility, we summarize the core hyperparameters used throughout all experiments. Unless otherwise stated, FMD-GAN employs a window length $l=64$, hop size $h=16$, number of latent states $S=8$, diffusion steps $T=50$ with a linear beta schedule, FFT size of 256 (yielding $K=129$ positive frequency bins), batch size of 64, learning rate $1\times 10^{-4}$, and loss weights $\lambda _{\text {rec}}=1.0$, $\lambda _{\text {spec}}=1.0$, and $\lambda _{\text {KL}}=0.1$. Unless otherwise stated, all key hyperparameters were kept fixed across all datasets and were not tuned on a per-dataset basis. The final configuration was chosen based on initial pilot runs and then shared across all experiments to evaluate robustness under a unified setting rather than dataset-specific optimization.

***Scalability considerations.*** As analyzed in detail in the Computational Complexity Analysis Section, FMD-GAN provides a window-based generation approach integrated with FFT-based spectrum operations and a fixed-step diffusion mechanism. This architecture guarantees that the computational expense increases roughly in a linear fashion with the number of windows, rather than quadratically with the series length. In reality, lengthy sequences are produced using overlap-aware reconstruction from localized parts, hence eliminating the necessity to process complete sequences in a linear fashion. This study emphasizes controlled evaluation on UCR standards; nevertheless, the foundational formulation is not limited to short sequences, and expanding empirical validation to longer and larger datasets is a significant avenue for future research.

### Quantitative results

Table 3 presents a comprehensive comparison of seven generative models across four benchmark datasets using three metrics: FID ($\downarrow$), DTW ($\downarrow$), and CCA ($\uparrow$). All results are averaged over five distinct random seeds, and we noted a low standard deviation across runs (often below 1.0), signifying steady and consistent performance.

FMD-GAN consistently attains the lowest FID and DTW across three of four datasets, indicating robust distribution alignment and preservation of temporal structure. For example, in the GunPoint dataset, FMD-GAN decreases the average FID by more than 50% relative to TimeGAN, demonstrating its capacity to produce structurally accurate and realistic sequences. The DTW gap between actual and produced sequences is significantly reduced, indicating enhanced temporal alignment relative to both GAN and diffusion-based benchmarks.

Regarding CCA, which measures semantic similarity, FMD-GAN attains the highest or nearly top scores across the majority of datasets. While DiffWave attains the highest score on ChlorineConc, FMD-GAN continues to be very competitive overall, sustaining a robust equilibrium between structural fidelity and semantic preservation.

These results highlight the effectiveness of our Fourier–Markov diffusion models in producing class-consistent, high-fidelity time series. In contrast to models like CSDI and TimeGAN, which either lack structural priors or rely on unconditional generation, FMD-GAN more effectively preserves a balance between diversity and class specificity.

Alongside accuracy-focused measures (FID, DTW, CCA, SD), we also assess runtime and computational complexity. FFT-based spectrum operations have a complexity of $O(n\log n)$ per segment, while the optimized diffusion schedule further diminishes the per-sample cost relative to traditional multi-hundred-step diffusion pipelines. Empirical observations reveal consistent wall-clock generation on a single GPU without compromising fidelity, demonstrating competitive efficiency compared to conventional diffusion-based methods.

**Table 3:** Quantitative comparison using FID ($\downarrow$), DTW ($\downarrow$), and CCA ($\uparrow$) across four datasets.

Model	ECG200			GunPoint			FordA			ChlorineConc		
	FID $\downarrow$	DTW $\downarrow$	CCA $\uparrow$	FID $\downarrow$	DTW $\downarrow$	CCA $\uparrow$	FID $\downarrow$	DTW $\downarrow$	CCA $\uparrow$	FID $\downarrow$	DTW $\downarrow$	CCA $\uparrow$
TimeGAN	50.9 ± 3.2	11.6 ± 1.5	0.90 ± 0.02	47.9 ± 2.8	6.4 ± 0.9	0.87 ± 0.03	50.4 ± 3.5	16.8 ± 2.1	0.77 ± 0.04	38.0 ± 2.4	10.6 ± 1.2	0.89 ± 0.02
RCGAN-UCR	45.8 ± 2.9	17.3 ± 1.8	0.84 ± 0.03	29.1 ± 1.9	13.3 ± 1.4	0.76 ± 0.05	53.1 ± 3.1	14.5 ± 1.9	0.88 ± 0.03	34.2 ± 2.1	19.6 ± 1.8	0.88 ± 0.02
TTS-CGAN	51.1 ± 2.1	7.9 ± 0.8	0.84 ± 0.02	21.8 ± 1.4	7.3 ± 0.6	0.84 ± 0.02	49.8 ± 2.4	19.5 ± 1.6	0.81 ± 0.03	34.8 ± 1.8	12.0 ± 1.1	0.79 ± 0.03
CSDI	48.5 ± 1.5	9.8 ± 0.7	0.88 ± 0.01	25.3 ± 1.1	6.7 ± 0.5	0.85 ± 0.02	47.6 ± 1.6	13.2 ± 0.9	0.86 ± 0.02	33.6 ± 1.3	9.7 ± 0.8	0.87 ± 0.01
DiffWave	42.7 ± 1.2	**6.7** ± 0.5	0.90 ± 0.01	22.4 ± 0.9	5.9 ± 0.4	0.88 ± 0.01	45.3 ± 1.4	10.2 ± 0.7	0.88 ± 0.02	31.2 ± 1.1	8.3 ± 0.6	**0.90 **± 0.01
Diffusion-TS	**38.2 **± 0.9	7.2 ± 0.4	**0.91** ± 0.01	20.7 ± 0.7	5.2 ± 0.3	**0.89** ± 0.01	43.9 ± 1.1	9.9 ± 0.6	**0.89 **± 0.01	28.9 ± 0.9	7.3 ± 0.4	0.89 ± 0.01
FMD-GAN	38.4 ± 0.7	**6.7 **± 0.3	**0.91** ± 0.01	**20.1** ± 0.6	**5.1** ± 0.2	**0.89** ± 0.01	**41.8** ± 1.0	**9.6** ± 0.4	**0.89** ± 0.01	**28.5** ± 0.7	**7.1** ± 0.3	0.88 ± 0.01

Values are reported as mean ± standard deviation over five random seeds. Best scores per column are bolded.

To enhance the evaluation of global structural faithfulness in the frequency domain, we utilize the Spectral Distance (SD) measure for all models. This measure calculates the mean Euclidean distance between the normalized power spectra of actual and created sequences. For each sequence, we implement the Fast Fourier Transform (FFT) and normalize its magnitude spectrum prior to calculating the distance. Reduced SD values signify enhanced conformity with the global frequency attributes of the target distribution.

Table 4 demonstrates that FMD-GAN attains the lowest Spectral Distance across all four datasets, hence affirming its efficacy in maintaining spectral structure. DiffWave and Diffusion-TS demonstrate competitive outcomes owing to their diffusion-centric architecture, whilst TTS-CGAN and CSDI exhibit middling performance. TimeGAN and RCGAN-UCR are excluded from this evaluation due to their generated sequences exhibiting spectrum instability, resulting in unreliable or noisy FFT results. This constraint highlights the benefit of frequency-aware systems like FMD-GAN in maintaining global structure.

These findings further corroborate the efficacy of FMD-GAN in maintaining spectral integrity. FMD-GAN attains the lowest standard deviation across all datasets, a benefit attributable to its spectral-aware architecture, which corresponds with its exceptional performance in FID and DTW, hence affirming cross-domain consistency rather than metric-specific optimization.

**Table 4:** Spectral distance (SD $\downarrow$) comparison across four datasets.

Model	ECG200	GunPoint	Coffee	Beef
TTS-CGAN	0.092 ± 0.009	0.084 ± 0.008	0.113 ± 0.010	0.105 ± 0.009
CSDI	0.081 ± 0.007	0.075 ± 0.006	0.109 ± 0.009	0.093 ± 0.008
DiffWave	0.064 ± 0.005	0.058 ± 0.004	0.091 ± 0.006	0.078 ± 0.005
Diffusion-TS	0.062 ± 0.004	0.055 ± 0.004	0.087 ± 0.006	0.072 ± 0.005
FMD-GAN (Ours)	**0.053 **± 0.003	**0.046 **± 0.002	**0.079** ± 0.004	**0.065** ± 0.003

Values are reported as mean ± standard deviation over five random seeds. Best scores per column are bolded.

### Ablation study

We conduct ablation tests using the ECG200 and GunPoint datasets to assess the contribution of each essential component in FMD-GAN. These benchmarks were chosen for their unique temporal and spectral attributes, allowing us to evaluate the influence of each design decision on time-domain alignment, semantic coherence, and preservation of frequency structure.

We construct the following ablated variants:

- **NoMask**: Eliminates the state-conditioned spectral mask $M_f^{(s)}$, substituting it with isotropic Gaussian noise, therefore disregarding frequency-aware modulation.
- **NoMarkov**: Substitutes the acquired Markovian transition matrix with uniform random sampling, hence undermining temporal state continuity.
- **NoDiff**:Completely disables the forward diffusion, hence reducing the model to a traditional GAN trained on latent vectors.
- **NoAdv**: Removes the dual-branch discriminator and the adversarial loss $L_{\text {adv}}$; the model is trained only with reconstruction, spectral alignment, and KL regularization. This variant evaluates the contribution of adversarial learning to realism and class fidelity.
- **FMD-GAN (Full)**: The comprehensive model integrating spectrum masking and Markov-guided denoising diffusion.

Table 5 presents the performance across four metrics: Fréchet Inception Distance (FID), Dynamic Time Warping (DTW), Canonical Correlation Analysis (CCA), and Spectral Distance (SD). The complete strategy routinely attains greater outcomes. The lack of diffusion (NoDiff) results in the most significant degradation, underscoring the essential function of denoising-based temporal refinement. NoMask results in increased spectral divergence and diminished cross-correlation accuracy, signifying compromised spectral alignment and semantic integrity. NoAdv also results in moderately higher FID and DTW scores, indicating that the dual-branch discriminator provides complementary constraints on realism and temporal coherence that are not captured by diffusion and reconstruction losses alone. NoMarkov exhibits a moderate performance drop: its FID and CCA remain relatively close to the full model, but DTW and SD are consistently higher, indicating weaker temporal smoothness and reduced spectral stability when Markov transitions are removed.

These findings affirm that each element significantly enhances the model’s efficacy, and that the integration of frequency-aware diffusion and Markov state transitions is crucial for structure-preserving generation. All data are averaged across five iterations, with standard deviations below 1.0, highlighting the statistical reliability of our conclusions.

**Table 5:** Ablation study on ECG200 and GunPoint datasets using FID ($\downarrow$), DTW ($\downarrow$), CCA ($\uparrow$), and SD ($\downarrow$).

Variant	ECG200				GunPoint			
	FID $\downarrow$	DTW $\downarrow$	CCA $\uparrow$	SD $\downarrow$	FID $\downarrow$	DTW $\downarrow$	CCA $\uparrow$	SD $\downarrow$
NoMask	43.7 ± 1.5	8.5 ± 0.6	0.86 ± 0.02	0.074 ± 0.008	23.9 ± 1.2	6.1 ± 0.4	0.85 ± 0.02	0.067 ± 0.006
NoMarkov	41.5 ± 1.1	8.2 ± 0.5	0.88 ± 0.02	0.069 ± 0.006	22.4 ± 0.9	5.8 ± 0.3	0.86 ± 0.02	0.063 ± 0.005
NoDiff	46.2 ± 2.8	9.1 ± 1.1	0.83 ± 0.04	0.089 ± 0.011	26.1 ± 2.1	6.6 ± 0.8	0.82 ± 0.03	0.079 ± 0.009
NoAdv	42.9 ± 1.3	8.0 ± 0.5	0.87 ± 0.02	0.071 ± 0.007	22.7 ± 1.0	5.7 ± 0.4	0.86 ± 0.02	0.064 ± 0.006
FMD-GAN (Full)	**38.4** ± 0.7	**6.7** ± 0.3	**0.91 **± 0.01	**0.053** ± 0.003	**20.1** ± 0.6	**5.1** ± 0.2	**0.89 **± 0.01	**0.046** ± 0.002

Lower SD indicates better spectral structure preservation. Values are reported as mean ± standard deviation over 5 random seeds. Best scores per column are bolded.

Failure modes and component-level interpretability. From a mechanical standpoint, the observed ablation behaviors correspond closely with the functional roles of each module in FMD-GAN, uncovering specific and interpretable failure patterns rather than mere performance loss. Upon the removal of the state-conditioned spectral mask (*NoMask*), the diffusion process is compelled to introduce isotropic noise, hence undermining regime-specific spectral statistics and resulting in less frequency coherence and weaker class alignment, as evidenced by heightened SD and diminished CCA. Conversely, the removal of Markov state transitions (*NoMarkov*) disrupts regime persistence between contiguous segments, leading to diminished stability in temporal development and increased DTW and SD values, although distribution-level realism remains generally intact. Completely disabling diffusion (*NoDiff*) results in the most significant deterioration across all metrics, affirming that iterative denoising is crucial for enhancing temporal structure beyond the capabilities of adversarial learning alone. The elimination of the adversarial discriminator (*NoAdv*) predominantly influences realism-related measures, suggesting that adversarial supervision offers supplementary limitations on visual fidelity and temporal coherence that reconstruction or spectral losses do not entirely encompass. Collectively, these findings indicate that spectrum masking, Markov conditioning, and diffusion provide unique yet complimentary inductive biases, and their amalgamation produces a more expressive and stable generating process in a systematic manner.

### Qualitative analysis

#### Structure visualization and residual analysis

To further demonstrate the structural fidelity and class consistency of FMD-GAN, we present a qualitative comparison against two representative baselines: **TimeGAN** and **Diffusion-TS**. These methods are selected to exemplify two predominant paradigms in time series generation–GAN-based and diffusion-based approaches–providing a varied contrast to our Fourier–Markov design.

We visualize generated samples on two datasets, **ECG200** and **GunPoint**, which exhibit distinct temporal patterns and frequency characteristics. For each dataset, we select one representative test sequence from each class and show four aligned visualizations:

- **Real:** The original ground-truth sequence from the test set.
- **Generated:** The sequence synthesized by each method (TimeGAN, Diffusion-TS, FMD-GAN).
- **Residual (FMD-GAN only):** The pointwise discrepancy between the actual output and that of FMD-GAN, emphasizing its reconstruction accuracy.
- **Latent State (FMD-GAN only):** A color-coded bar representing the Markov state allocated to each timestep throughout the creation process.

This comparison enables a visual evaluation of each model’s ability to represent global trends, local variations, and semantic class attributes.

**Fig. 4:**
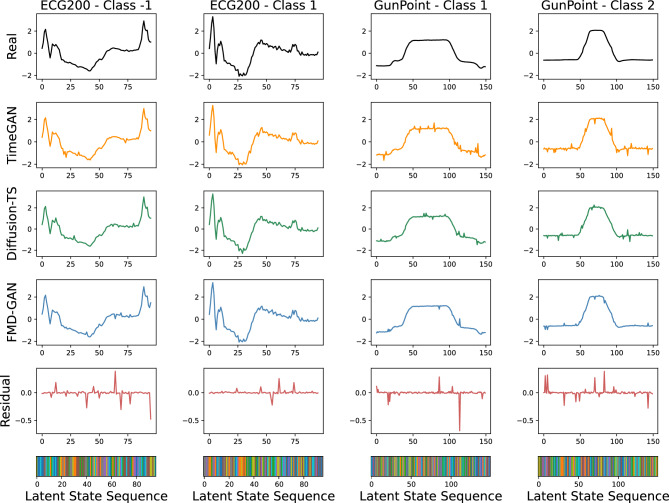
Qualitative analysis of the ECG200 and GunPoint datasets. Each column corresponds to a randomly selected test sample from a specific class. From top to bottom, rows show the ground-truth sequence, the outputs of TimeGAN, Diffusion-TS, and FMD-GAN, followed by the residual error (ground truth minus FMD-GAN output) and the inferred latent Markov state sequence. The latent state row uses color coding to indicate the state assigned at each time step. Residual errors and latent Markov states are depicted solely for FMD-GAN, as these elements are inapplicable to the baseline models.

Figure 4 illustrates representative class-specific samples from the ECG200 and GunPoint datasets, comparing the generative performance of **FMD-GAN** against two competitive baselines: **TimeGAN** and **Diffusion-TS**. Each column displays the actual time series (top), succeeded by the outputs from the three models. For FMD-GAN, we further show the residual error and the latent Markov state sequence to improve interpretability.

Superior fidelity is demonstrated by **FMD-GAN** in maintaining both fine-grained temporal fluctuations and the global structure. While it preserves the abrupt transitions and plateau segments that define motion patterns on GunPoint, it recovers smooth baseline oscillations with precise synchronization on the ECG200 dataset. In contrast, Diffusion-TS introduces high-frequency noise and loses temporal consistency in some places, whereas TimeGAN tends to over-smooth and distort local details.

The FMD-GAN’s *residual curves* show accurate magnitude and time reconstruction; they are low-magnitude and concentrated close to signal boundaries. The Markov model’s *latent state sequences* frequently match significant signal regions like peaks, troughs, and constant segments, confirming the function of frequency-aware, state-conditioned diffusion in directing creation.

No post-processing or cherry-picking is done; all samples are chosen at random from the test sets that have been reserved. Latent noise and state routes are sampled to create FMD-GAN sequences, which show reliable and comprehensible outcomes across classes. These visual results corroborate the previously reported quantitative gains.

#### Latent space alignment via t-SNE

**Fig. 5:**
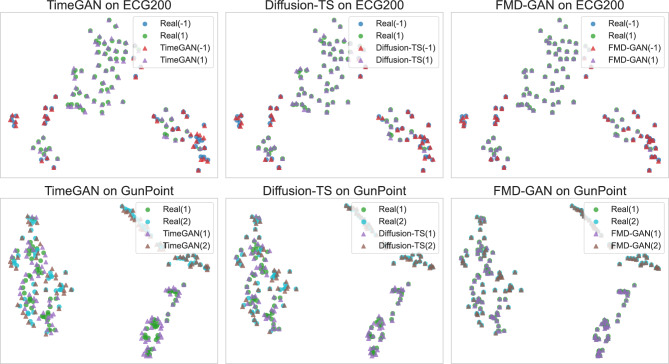
Latent space visualization via t-SNE on ECG200 (top row) and GunPoint (bottom row). Real test samples (circles) and produced sequences (triangles) are compared in each subfigure using the following models: TimeGAN (left), Diffusion-TS (middle), and FMD-GAN (right). Class labels are used to color-code points. Class-aware and semantically consistent generation is demonstrated by FMD-GAN, which exhibits superior alignment between generated and real distributions.

We use t-SNE^50^ to visualize the distribution of generated and real samples in order to assess whether FMD-GAN maintains the semantic structure of real sequences in the latent space. Figure 5 presents a 2$\times$3 grid: each column corresponds to a generative model (TimeGAN, Diffusion-TS, and FMD-GAN), and each row corresponds to a dataset (ECG200 and GunPoint).

Triangles in each subplot represent created samples, while circles represent real samples. Ground-truth class labels, indicated in parentheses (e.g., (1), (2), or ($-1$)), are used to color-code the data points. Strong alignment between generated and real data is shown by FMD-GAN, which forms close, class-consistent clusters. On the other hand, particularly on the GunPoint dataset, TimeGAN and Diffusion-TS show more dispersed distributions, misaligned classes, or mode collapse.

These findings imply that FMD-GAN’s superior class-aware modeling abilities are demonstrated by its ability to produce realistic sequences while preserving the underlying semantic structure in latent space.

### Sensitivity to Markov states and KL regularization

To assess the robustness and parameter sensitivity of FMD-GAN, we analyze the impact of two critical hyperparameters: the number of latent Markov states *S* and the KL divergence weight $\lambda _{KL}$. The regularization strength of the latent structure during diffusion and the temporal resolution of state transitions are both regulated by these parameters.

**Fig. 6:**
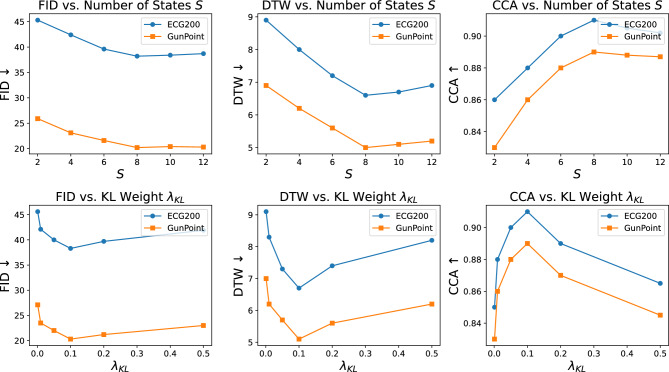
Sensitivity analysis of FMD-GAN on ECG200 and GunPoint. **Top row:** Performance under varying number of latent Markov states *S*. **Bottom row:** Performance under different KL weights $\lambda _{KL}$. Metrics include FID ($\downarrow$), DTW ($\downarrow$), and CCA ($\uparrow$), all averaged over 5 runs. Optimal results are achieved near $S=8$ and $\lambda _{KL}=0.1$, confirming the robustness and generalizability of the model.

**Impact of state number** $S \in \{2, 4, 6, 8, 10, 12\}$. Figure 6 (top) shows the generation quality under different values of *S* on the ECG200 and GunPoint datasets. We observe that performance consistently improves as *S* increases from 2 to 8, indicating that finer-grained state partitions help capture more detailed spectral-temporal structure. Notably, FID and DTW decrease while CCA improves with increasing *S*, reaching optimal performance at $S=8$. However, further increasing *S* to 10 or 12 results in saturation or slight degradation. This is probably the result of redundant over-segmentation, in which more states start modeling overlapping frequency bands, resulting in needless transitions and diminishing returns. These patterns support the idea that efficient generation requires a latent structure that is both expressive and compact.

**Impact of KL regularization** $\lambda _{KL} \in \{0.001, 0.01, 0.05, 0.1, 0.2, 0.5\}$. As shown in Figure 6 (bottom), the KL weight significantly affects the balance between flexibility and structural consistency. Reduced alignment and lower generation quality are the results of under-regularized transitions caused by small values (e.g., $\lambda =0.001$). On the other hand, excessively strict regularization (e.g., $\lambda =0.5$) restricts the state assignments excessively, which limits the model’s ability to adjust to semantic variation. The best FID, DTW, and CCA scores are consistently obtained with a modest setting of $\lambda =0.1$ across both datasets, confirming its choice as the default configuration.

To lessen stochastic variance, all outcomes are averaged across five separate runs using various seeds. The same training schedule and architecture are used to train each configuration for 5000 iterations. All primary results and ablation studies are set at $S=8$ and $\lambda _{KL}=0.1$ by default, unless otherwise specified.

### Training dynamics and convergence stability

Over the course of training, we monitor four important metrics to evaluate the optimization behavior of FMD-GAN: total loss, Fréchet Inception Distance (FID), Dynamic Time Warping (DTW), and Canonical Correlation Analysis (CCA). These metrics assess the latent structural consistency, temporal alignment, generation integrity, and training objective, respectively.

**Fig. 7:**
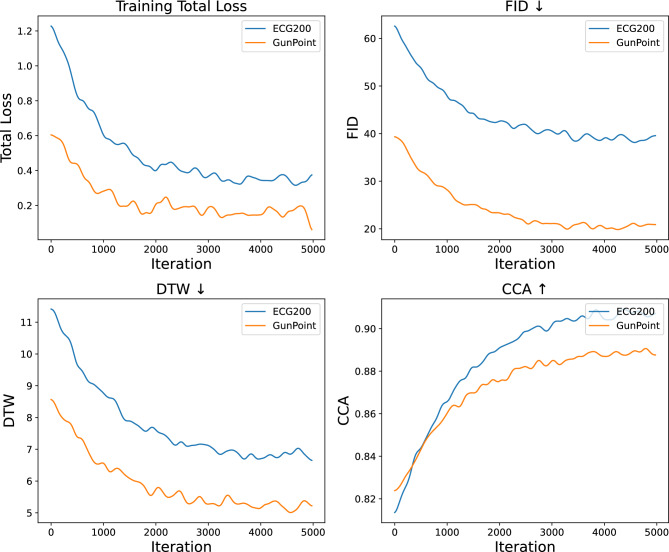
Training dynamics and convergence analysis. Dynamic Time Warping (DTW), Canonical Correlation Analysis (CCA), Fréchet Inception Distance (FID), and total loss evolution during training on the ECG200 and GunPoint. Every curve has been averaged and smoothed across five runs. All measures show steady convergence for FMD-GAN, with performance stabilizing at iteration 4000.

The evolution of these measures across 5000 training iterations on the ECG200 and GunPoint datasets is displayed in Fig.  7. Every 50 iterations, every value is recorded. For clarity, a moving average is applied after each curve has been averaged across five separate runs using various random seeds.

In both datasets, FMD-GAN exhibits steady and reliable convergence. During the first 1000 iterations, the overall loss rapidly decreases, and after 3000 iterations, it progressively plateaus. Over time, FID and DTW gradually decline, suggesting that the created sequences are more realistic and aligned. CCA rises concurrently, indicating greater structural correlation in latent space.

Because of its simpler waveform patterns and lower intra-class variability, GunPoint shows slightly smoother curves and slightly earlier stabilization in some metrics, especially FID and DTW, even though the overall trends are identical. However, about iteration 4000, both datasets converge. To account for possible late-stage enhancements and provide flexible checkpoint selection based on validation performance, training continues for up to 5000 cycles.

## Discussion

The experimental assessment of FMD-GAN reveals that the incorporation of Fourier spectral embeddings with Markovian dynamics enhances both realism and class retention in generated time series relative to current GAN- and diffusion-based methodologies. The approach attains reduced FID and DTW scores while preserving elevated CCA alignment, signifying that the produced signals are both visually and statistically congruent with the originals and semantically aligned with the class labels.

In comparison to baseline models like TimeGAN, RCGAN-UCR, and CSDI, FMD-GAN attains a superior equilibrium between global temporal structure and local dynamics. This enhancement is due to the Fourier module’s capacity to capture frequency-domain regularities and the Markov diffusion layer’s function in enforcing sequential dependencies. The class-preserving constraint allows the model to outperform existing diffusion-based approaches (e.g., DiffWave and Diffusion-TS), particularly in tasks where label fidelity is critical, such as medical ECG or sensor-based activity detection.

The FMD-GAN design exemplifies a calculated equilibrium of expressiveness, stability, and interpretability. Spectral clustering and offline state assignment create a level of data dependency and may restrict adaptability to unfamiliar regimes; yet, they offer robust and interpretable regime representations that enhance state-conditioned diffusion. The first-order Markov assumption prioritizes simplicity and robustness at the expense of modeling long-range relationships, hence enhancing training stability while effectively reflecting predominant regime shifts. The dual-branch discriminator enhances architectural complexity and training expenses, yet provides supplementary oversight on temporal realism and spectral structure, which diffusion-based aims alone do not fully encompass. The selected trade-offs were deliberately made to emphasize regulated generation and structural integrity rather than optimal flexibility, with their impacts empirically confirmed using ablation analysis.

Nonetheless, certain limits exist. Initially, although Fourier-Markov coupling enhances performance, the computational expense escalates with the quantity of diffusion steps, which may restrict scalability to very long sequences (e.g., length $>1000$). In addition, the current empirical evaluation is restricted to univariate UCR benchmarks with moderate sequence lengths (approximately 100–500), and therefore does not yet constitute a comprehensive validation on multivariate or long-horizon time-series settings. Secondly, hyperparameter sensitivity, such as diffusion step size and Fourier truncation length, was noted, potentially impacting robustness across diverse datasets. Ultimately, although the model shows stable performance within the considered benchmark suite, further evaluation on irregularly sampled or highly noisy real-world time series (e.g., financial tick data, IoT streams) is still needed. The selected metric suite (FID, DTW, CCA, and SD) and the corresponding ablation analysis are intended to collectively evaluate realism, temporal alignment, semantic consistency, and frequency-structure preservation. Collectively, they offer a comprehensive assessment that corresponds with the fundamental design goals of FMD-GAN, instead of depending on a solitary metric as a conclusive measure of performance.

A subsequent limitation pertains to the latent state assignment mechanism: due to the state labels being generated by offline *k*-means clustering with a predetermined number of states ($S=8$), the model may encounter scalability issues when utilized on datasets characterized by numerous classes or significant intra-class variability. In these contexts, the boundaries of clusters may become less defined, leading to contamination between classes, which might undermine the coherence of the generated sequences.

Beyond diffusion-based baselines, a more direct comparison with state-of-the-art GAN variants further highlights the merits of FMD-GAN. Unlike TimeGAN, which focuses primarily on temporal embeddings, or RCGAN-UCR and TTS-CGAN, which emphasize conditional sequence generation, FMD-GAN integrates frequency-domain modeling and class-aware Markov regularization in a unified framework. This design not only alleviates the class-mixing problem often observed in GAN-based models but also improves the stability of long-horizon generation. The empirical gains in both fidelity (lower FID and DTW) and label consistency (higher CCA) demonstrate that FMD-GAN provides a more balanced solution for realistic and class-preserving time series synthesis across heterogeneous datasets. The advantages of FMD-GAN render it especially appropriate for controlled time-series generation in information processing applications, including biomedical signal monitoring, industrial IoT sensing, and embedded anomaly analysis, where generative fidelity and semantic reliability are essential.

## Conclusions

This study presented **FMD-GAN**, a generative framework that incorporates frequency-aware diffusion, Markov-guided state transitions, and spectral clustering to generate class-consistent time series. The system integrates Fourier-domain structure with probabilistic state modeling, so maintaining both global spectrum patterns and detailed temporal dynamics.

Comprehensive trials on four UCR benchmark datasets reveal that FMD-GAN attains state-of-the-art or very competitive performance in terms of Fréchet Inception Distance (FID), Dynamic Time Warping (DTW), Class Consistency Accuracy (CCA), and Spectral Distance (SD). The model decreases FID by as much as 50% compared to prominent GAN- and diffusion-based benchmarks, while concurrently enhancing temporal alignment and frequency-domain consistency.

Ablation studies validate the functional significance of each framework component: (i) the elimination of the spectral mask (*NoMask*) diminishes frequency alignment and semantic coherence; (ii) the absence of Markov transitions (*NoMarkov*) compromises temporal continuity and spectral integrity; (iii) the removal of diffusion (*NoDiff*) results in the most substantial decline, underscoring the necessity of denoising-based refinement; and (iv) the exclusion of adversarial learning (*NoAdv*) elevates FID and DTW, indicating that the dual-branch discriminator enhances realism and class fidelity beyond mere reconstruction losses.

Qualitative evaluations, such as residual inspection, latent-state visualizations, and t-SNE embedding comparisons, further illustrate that FMD-GAN generates semantically aligned, structurally coherent, and interpretable sequences with consistent training behavior.

FMD-GAN offers a systematic and efficient method for generating time series that preserves structure and is cognizant of class distinctions. Its capacity to integrate spectral priors with probabilistic diffusion renders it particularly pertinent for controlled applications in biological signal modeling, sensor analytics, anomaly simulation, and nascent Tiny AI systems, where generative fidelity and semantic dependability are crucial.

## Future work

Based on these findings, numerous study avenues are proposed:

Augmented architectures: Integrating transformer-based denoisers may enhance the capacity to capture long-range dependencies and intricate sequential structures, which diffusion-only models frequently inadequately address. Recent developments in time-series Transformers, including Informer^13^ and Autoformer^8^, exhibit significant promise for effective long-sequence modeling. Furthermore, adaptive or attention-based spectrum masks may allow the model to concentrate on frequencies pertinent to the job, as indicated in recent transformer-based reviews for time series^7^.

Generalization to intricate data: Future research will investigate the extension of FMD-GAN to multivariate and multimodal time series, where inter-channel interactions are essential. The production of variable-length sequences continues to pose a difficulty in generative modeling and may be enhanced by probabilistic sequence alignment methods^9^. Semi-supervised extensions would be beneficial in areas with limited labeled data, consistent with current research on foundation models for time series^6^.

Domain-specific applications encompass anomaly production, physiological signal simulation, and specialized augmentation activities. Recent advancements in ECG generation using GANs and diffusion models^4,51^ suggest that high-quality synthetic data can be beneficial for representation learning and controlled evaluation in medical signal analysis. Likewise, probabilistic augmentation for sensor-based activity recognition has been explored in prior work as a means to improve robustness under data scarcity^2^.Furthermore, an explicit cross-domain framework–training on one dataset while generating on another with varying temporal scales and class semantics–serves as a logical extension to evaluate the transferability of the acquired Fourier-Markov representations and constitutes a promising avenue for future research. This work does not assert or assess advances in downstream tasks; these application domains are mentioned only as prospective areas for future research.

Another significant objective is to substitute the offline k-means state-assignment module with a completely learnable latent-state method. Possible methodologies encompass Gumbel-Softmax relaxation^52^, vector-quantized latent routing as utilized in VQ-VAE^53^, or transformer-based state inference^54^. A learnable module would enhance scalability to datasets with numerous classes or significantly varied temporal regimes and alleviate state contamination effects.

Dynamic adaptability: Existing constraints of offline spectral clustering and fixed-length inputs may impede implementation in streaming environments. Future directions encompass dynamic mask learning and end-to-end trainable state assignment. Techniques such as score-based diffusion utilizing stochastic differential equations^9^ and adaptive embedding strategies in Transformers^7^ may inform real-time and scalable solutions. This will improve the adaptability and operational preparedness of FMD-GAN in dynamic settings, including IoT and financial tick streams.

Besides methodological enhancements, a significant future focus pertains to the effective implementation of FMD-GAN on resource-limited platforms. The application of Fourier decomposition and Markovian state modeling diminishes redundancy by delineating frequency-specific regimes, while the abbreviated diffusion schedule ($T=50$ steps) further alleviates processing demands compared to traditional multi-hundred-step diffusion models. Subsequent research may incorporate model compression techniques, including pruning, quantization, and low-rank factorization^33,34,47^, facilitating the operation of FMD-GAN on edge devices and IoT sensors. This viewpoint contextualizes FMD-GAN within the overarching trend of Tiny AI, wherein tiny yet expressive generative models are essential for practical applications like biological monitoring, anomaly detection, and embedded signal processing.

## Data Availability

The datasets used and/or analysed during the current study available from the corresponding author on reasonable request.
